# Updated Estimates of Chronic Conditions Affecting Risk for Complications from Coronavirus Disease, United States

**DOI:** 10.3201/eid2609.202117

**Published:** 2020-09

**Authors:** Mary L. Adams, David L. Katz, Joseph Grandpre

**Affiliations:** On Target Health Data LLC, Suffield, Connecticut, USA (M.L. Adams);; True Health Initiative, Derby, Connecticut, USA (D.L. Katz);; Wyoming Department of Health, Cheyenne, Wyoming, USA; (J. Grandpre)

**Keywords:** population-based estimates, chronic conditions, risk factors, complications, coronavirus disease, COVID-19, severe acute respiratory syndrome coronavirus 2, SARS-CoV-2, infection, viruses, respiratory infections, zoonoses, United States

## Abstract

We updated estimates of adults at risk for coronavirus disease complications on the basis of data for China by using recent US hospitalization data. This update to our previous publication substitutes obesity for cancer as an underlying condition and increases adults reporting any of the conditions from 45.4% to 56.0%.

We had earlier reported estimates of any conditions affecting risk for complications from coronavirus disease (COVID-19) (hypertension, cancer, asthma, chronic obstructive pulmonary disease, diabetes, and cardiovascular disease) on the basis of mortality data from China (*1*). Recent US data on hospitalizations for COVID-19 (*2*) indicate that 89% had an underlying condition, but obesity was substituted for cancer in that list. We update those previous estimates of adults at risk (*1*) by using the US hospitalization data definition, which includes obesity instead of cancer.

## The Study

Methods were described earlier (*1*) by using publicly available 2017 Behavioral Risk Factor Surveillance System data, which excludes nursing homes and prisons (*3*). Our key variable included adults reporting they were ever told they had diabetes, asthma (and still had it), chronic obstructive pulmonary disease, hypertension, cardiovascular disease (including heart disease or stroke) or they were obese with body mass index >30.0 kg/m^2^ based on self-reported height and weight. Adults who reported >1 of the conditions were considered to be at risk for hospitalization for COVID-19 because of an underlying condition. New measures included the risk factors of ever smoking 100 cigarettes; sedentary lifestyle, defined as no leisure time physical activity in the past month; and inadequate fruit and vegetable consumption, defined as consuming the combination <5 times/day on the basis of responses to 5 questions. A measure of the number of risk factors (0–3) was also created. Demographic measures were the same as in the previous report (*1*) with the addition of census region (Northeast, Midwest, South, or West) (*4*).

Stata version 14.1 (StataCorp LP, https://www.stata.com) was used for analysis to account for the complex sample design of the Behavioral Risk Factor Surveillance System. Point estimates and 95% CIs are reported by using the weights, stratum, and PSU variables supplied in the dataset (*3*). Missing values were excluded from analysis.

The sample was described previously (*1*). Prevalence rates of the separate conditions were 8.5% for cardiovascular disease, 6.6% for chronic obstructive pulmonary disease, 9.1% for asthma, 10.8% for diabetes, 32.4% for hypertension, and 30.1% for obesity. We provide updated results (Table 1) based on US hospitalizations, along with the results previously published (*1*), with the difference being the substitution of obesity for cancer as an underlying condition. Overall, 56.0% (95% CI 55.7%–56.4%) of respondents had >1 underlying conditions using the updated definition, compared with 45.4% using the previous definition, an increase of 23% over the previous rate. For the updated measure, 49.2% of employed or self-employed adults reported an underlying condition, and 18.7% of employed adults reported >2 underlying conditions. These results compare with 26.6% of all adults reporting >2 underlying conditions. Although the percentage of adults with any of the conditions increased with age (Table 1), 60.7% of those with underlying conditions were <60 years of age when using the new definition, compared with 53.4% when using the earlier definition.

**Table 1:** Comparison of population demographics for 2 studies of chronic conditions affecting risk for complications from coronavirus disease, United States*

Population group	Previous %	Previous 95% CI	Updated %	Updated 95% CI	Updated sample size
Total	45.4	45.1–45.7	56.0	55.7–56.3	398,865
Sex					
M	45.4	44.9–45.9	56.7	56.2–57.2	183,097
F	45.4	45.0–45.9	55.3	54.8–55.8	215,768
Age, y					
18–29	19.8	19.1–20.4	33.7	32.9–34.5	42,434
30–39	26.8	26.0–27.5	45.0	44.2–45.9	44,676
40–49	38.1	37.3–39.0	53.6	52.7–54.5	49,449
50–59	55.1	54.3–55.9	63.4	62.6–64.1	73,839
60–69	68.0	67.3–68.6	71.4	70.8–72.1	90,088
70–79	79.5	78.7–80.2	78.8	78.0–79.6	63,627
>80	80.7	79.5–81.8	79.1	77.9–80.2	31,915
Race/ethnicity					
White (non-Hispanic)	48.0	47.7–48.4	56.7	56.3–57.0	307,282
Black	52.1	51.1–53.1	65.8	64.7–66.8	31,939
Hispanic	35.5	34.5–36.5	52.3	51.2–53.5	26,837
American Indian/Alaska Native	55.5	52.9–58.1	67.7	65.3–70.1	7,468
Asian/Pacific Islander	27.8	25.9–29.7	34.1	32.1–36.3	9,108
Other	46.5	44.5–48.4	57.0	55.0–59.0	10,089
Employment					
Employed/self-employed	35.4	35.0–35.8	49.2	48.7–49.6	201,050
Out of work	47.0	45.5–48.5	59.2	57.7–60.7	17,003
Homemaker	39.6	38.2–41.0	52.7	51.1–54.3	19,710
Student	18.1	16.9–19.4	28.1	26.6–29.7	10,804
Retired	75.7	75.1–76.3	76.3	75.6–76.9	119,720
Unable to work	79.3	78.3–80.3	84.5	83.6–85.4	28,472
Insurance status					
Insured	47.1	46.8–47.5	56.9	56.5–57.2	367,956
Uninsured	33.4	32.4–34.4	50.1	48.9–51.2	29,804
State					
AL	54.2	52.6–55.9	65.6	63.9–67.3	6,083
AK	43.6	40.7–46.5	56.5	53.4–59.5	2,961
AZ	44.6	43.6–45.6	55.5	54.4–56.6	13,824
AR	53.3	50.7–55.8	65.1	62.5–67.7	4,713
CA	41.0	39.6–42.4	50.2	48.7–51.7	8,304
CO	40.0	38.8–41.2	47.6	46.3–48.9	8,812
CT	45.0	43.7–46.3	54.0	52.5–55.4	9,302
DE	48.7	46.5–50.9	59.2	56.9–61.5	3,554
DC	38.3	36.4–40.1	45.6	43.5–47.6	4,012
FL	46.9	45.4–48.5	57.4	55.7–59.0	19,402
GA	45.6	44.0–47.2	57.3	55.5–59.1	5,247
HI	44.0	42.5–45.5	52.5	50.9–54.1	7,226
ID	42.6	40.7–44.5	53.3	51.2–55.3	4,535
IL	44.9	43.3–46.6	56.8	55.1–58.6	5,208
IN	48.7	47.5–49.8	59.7	58.5–60.9	12,676
IA	44.9	43.6–46.2	58.6	57.2–59.9	6,991
KS	45.9	45.1–46.8	58.0	57.1–58.9	19,304
KY	53.2	51.5–55.0	62.7	60.9–64.5	7,826
LA	51.6	49.7–53.5	61.9	60.0–63.9	4,328
ME	49.9	48.3–51.5	58.4	56.8–60.0	9,141
MD	44.9	43.5–46.2	56.4	55.0–57.9	12,056
MA	43.4	41.5–45.4	52.1	50.1–54.2	6,086
MI	49.4	48.2–50.6	59.4	58.1–60.6	9,863
MN	38.8	37.9–39.7	49.9	48.9–50.9	15,155
MS	51.9	49.8–54.0	63.3	61.1–65.5	4,627
MO	46.2	44.6–47.8	57.9	56.2–59.5	6,828
MT	44.0	42.3–45.8	51.4	49.5–53.3	5,409
NE	43.4	42.2–44.6	56.6	55.3–57.9	14,059
NV	46.1	43.7–48.5	55.9	53.3–58.4	3,427
NH	45.9	44.0–47.8	54.5	52.4–56.6	5,077
NJ	45.6	44.0–47.1	55.2	53.5–56.8	10,160
NM	45.3	43.5–47.0	56.1	54.3–58.0	5,908
NY	42.2	41.0–43.4	51.5	50.2–52.8	10,937
NC	47.5	45.6–49.3	59.2	57.2–61.1	4,386
ND	42.0	40.5–43.6	55.4	53.7–57.1	6,430
OH	48.3	46.9–49.6	59.4	58.0–60.8	11,138
OK	50.7	49.0–52.3	62.7	61.0–64.4	5,891
OR	44.7	43.1–46.3	54.7	53.0–56.4	4,757
PA	47.8	46.2–49.5	57.3	55.6–59.0	6,022
RI	47.8	45.9–49.8	56.9	54.8–58.9	4,987
SC	49.9	48.6–51.3	61.6	60.2–63.0	10,191
SD	43.3	41.2–45.5	55.2	52.9–57.5	6,382
TN	51.1	49.3–53.0	61.1	59.2–63.0	5,215
TX	43.7	41.8–45.5	56.5	54.5–58.4	10,972
UT	37.3	36.2–38.5	47.4	46.1–48.7	9,115
VT	45.8	44.1–47.5	54.2	52.4–56.0	5,852
VA	45.2	43.8–46.6	55.8	54.3–57.3	8,630
WA	44.0	42.9–45.1	53.6	52.4–54.8	11,694
WV	58.7	57.0–60.4	68.8	67.1–70.5	4,999
WI	43.9	42.1–45.7	55.4	53.5–57.3	5,269
WY	44.0	42.2–45.9	53.6	51.7–55.6	4,084
Census region					
Northeast	NI	NI	54.0	53.3–54.8	67,564
Midwest	NI	NI	57.4	56.9–58.0	119,303
South	NI	NI	58.9	58.3–59.5	122,132
West	NI	NI	51.6	50.8–52.4	90,056
Risk factors					
Ever smoked					
Yes	NI	NI	64.3	63.8–64.8	170,149
No	NI	NI	50.5	50.0–51.0	219,779
Sedentary lifestyle					
Yes	NI	NI	67.8	67.2–68.5	100,438
No	NI	NI	52.3	51.9–52.7	276,037
Eat <5 times/day					
Yes	NI	NI	57.0	56.6–57.4	300,938
No	NI	NI	50.0	49.0–50.9	58,286
No. risk factors					
0	NI	NI	42.8	41.6–44.0	31,529
1	NI	NI	48.9	48.4–49.5	146,808
2	NI	NI	62.1	61.5–62.7	133,833
3	NI	NI	73.2	72.3–74.1	43,147

*Previous results were based on mortality data from China (*1*), and underlying conditions include cardiovascular disease, diabetes, chronic obstructive pulmonary disease, asthma, hypertension, or cancer other than skin. Updated results are based on US hospitalization data (*2*), and obesity is substituted for cancer as an underlying condition. Sample size in previous study was slightly larger than that for the updated study. NI, results of analysis that were not included in the original study.

Prevalence rates for the risk factors were 40.4% for ever smoking, 26.6% for sedentary lifestyle, and 84.1% for inadequate fruit and vegetable consumption. Adults with each risk factor (or more risk factors) were more likely than those without the risk factor (or fewer) to report any underlying condition (Table 1). Among ever smokers, 40.7% currently smoke, resulting in a current smoking rate of 16.5%. Updated state rates ranged from 45.6% in the District of Columbia to 68.8% in West Virginia, which was several percentage points higher but over a similar range of states compared with earlier estimates. State results obtained directly from Stata (Table 2) list the number of adults in each state at increased risk for hospitalizations and the percentage that number represents among all states, again showing both previously published and updated results.

**Table 2:** Comparison of number of adults at risk for 2 studies of chronic conditions affecting risk for complications from coronavirus disease, United States*

State	Previous results			Updated results	
	No. adults at risk	% Total		No. adults at risk	% Total
AL	1,997,864	1.78		2,252,938	1.53
AK	237,208	0.21		291,023	0.23
AZ	2,351,799	2.10		2,655,251	2.13
AR	1,181,105	1.06		1,325,336	0.91
CA	12,240,142	10.93		13,490,767	11.97
CO	1,701,776	1.52		1,876,647	1.76
CT	1,239,597	1.11		1,329,957	1.1
DE	357,530	0.32		389,066	0.29
DC	213,357	0.19		233,818	0.23
FL	7,696,749	6.88		8,296,062	6.44
GA	3,541,358	3.16		3,974,293	3.09
HI	486,156	0.43		543,222	0.46
ID	534,533	0.48		631,470	0.53
IL	4,404,556	3.93		5,243,320	4.11
IN	2,428,188	2.17		2,790,038	2.08
IA	1,067,133	0.95		1,287,375	0.98
KS	983,323	0.88		1,124,218	0.86
KY	1,789,444	1.60		1,963,497	1.39
LA	1,806,330	1.61		1,996,887	1.44
ME	530,809	0.47		594,575	0.45
MD	2,054,758	1.84		2,323,720	1.83
MA	2,302,809	2.06		2,511,936	2.15
MI	3,749,235	3.35		4,175,950	3.13
MN	1,625,778	1.45		1,882,783	1.68
MS	1,150,036	1.03		1,306,805	0.92
MO	2,137,650	1.91		2,466,200	1.9
MT	356,113	0.32		387,405	0.34
NE	616,905	0.55		743,149	0.59
NV	1,048,591	0.94		1,167,736	0.93
NH	485,340	0.43		522,567	0.43
NJ	3,106,880	2.78		3,265,515	2.64
NM	701,585	0.63		804,730	0.64
NY	6,419,321	5.73		7,093,023	6.13
NC	3,713,582	3.32		4,186,911	3.15
ND	243,096	0.22		298,972	0.24
OH	4,268,748	3.81		4,881,408	3.66
OK	1,461,941	1.31		1,665,703	1.18
OR	1,418,689	1.27		1,573,562	1.28
PA	4,738,414	4.23		5,340,392	4.15
RI	393,069	0.35		426,186	0.33
SC	1,912,134	1.71		2,189,219	1.58
SD	281,110	0.25		333,818	0.27
TN	2,610,800	2.33		2,863,321	2.09
TX	8,977,387	8.02		10,449,000	8.24
UT	796,721	0.71		915,321	0.86
VT	226,397	0.20		246,641	0.20
VA	2,921,171	2.61		3,295,289	2.63
WA	2,464,452	2.20		2,711,697	2.25
WV	827,193	0.74		907,986	0.59
WI	1,949,872	1.74		2,269,999	1.82
WY	194,544	0.17		220,916	0.18
Total	111,943,278	100		125,717,620	100

*Previous results were based on mortality data from China (*1*), and underlying conditions include cardiovascular disease, diabetes, chronic obstructive pulmonary disease, asthma, hypertension, or cancer other than skin. Updated results are based on US hospitalization data (*2*), and obesity is substituted for cancer as an underlying condition.

## Conclusions

We estimate that 56.0% of US adults, with a wide range across age groups and states, have >1 underlying conditions that increase risk for hospitalization caused by COVID-19. Our previous estimate of adults at risk for complications from COVID-19 (*1*) that included cancer and excluded obesity based on data for China, where obesity is much less prevalent (*5*), resulted in an estimate of 45.4%. Thus, the 23% increase appears to be the result of including as an underlying condition. In addition, although rates increase with increasing age group, over 60% of adults with underlying conditions by this new definition are <60 years of age. The underlying condition rate of 49.2% among the employed has potential implications as persons return to work. Also, the 26.6% of all adults who reported >2 underlying conditions, including 18.7% of the employed, might be at even greater risk for hospitalization based on results from other studies (*6*). Taken together, these results suggest that risk stratification based on age or number of underlying conditions might be considered as a means of more safely phasing in returning to work.

All the underlying conditions used in our updated measure are conditions for which behavioral risk factors have been well established (*7**,**8*). In unadjusted results each selected risk factor was associated with increased likelihood of reporting any of the 6 underlying conditions. In addition, the results for increasing number of risk factors indicate a stepwise increase in the percentage of adults reporting any of the underlying conditions with each additional risk factor (Table 1). These results suggest the potential for lowering risk for COVID-19 hospitalizations by reducing any or all of these 3 risk factors. In addition, being older, male, or African American also increased the likelihood of reporting an underlying condition, which are all groups that hospitalization data (*2*) suggested were disproportionately affected by COVID-19. These results suggest that observation might be caused by increased rates of underlying conditions among these groups. In addition, living in either the Midwest or South increased the likelihood of reporting an underlying condition compared with living in the West. This result is consistent with studies showing that obesity rates are also highest in these regions (*9*).

Our study does not address possible differences in contracting the disease, only the risk for hospitalization among those with COVID-19, based on US results for underlying conditions (*2*). Because only noninstitutionalized adults were surveyed, 1.3 million adults in nursing homes (*10*) were excluded, which almost certainly underestimates those with underlying conditions who were included in hospitalization data. Data are self-reported, and reliability and validity can vary for different measures tested (*11*). However, as long as a respondent was told they had a chronic condition, validity has been shown to be high. Age groups used for analysis did not match those used for weighting data, but that factor should have a minimal effect on results. Low response rates could introduce bias but, as noted, validity appears high for most measures used in this study.

We estimate 56.0% of US adults are at risk for needing hospitalization for COVID-19 because of underlying conditions, representing a 23% increase from the 45.4% earlier estimates, which excluded obesity. These underlying conditions are, in turn, associated with modifiable risk factors, including ever smoking, being sedentary, and inadequate fruit and vegetable consumption. These results suggest the potential for policies for opening businesses based on risk stratification of the population and for possible improvement of risk status through lifestyle change. A national focus on, and support for, a health promotion campaign would be timely.
